# Supplementary material for: Role of Adiponectin and Brain Derived Neurotrophic Factor in Metabolic Regulation Involved in Adiposity and Body Fat Browning
Source: J Clin Med. 2020 Dec 26;10(1):56. doi: 10.3390/jcm10010056 (PMC7794779; doi:10.3390/jcm10010056)
Supplement: Supplementary file 1 [file jcm-10-00056-s001.pdf]

Table S1. Fat browning primers

	Fw	Rev
Prdm16	5'-CGTCCATCGCGGAGAAATAC-3'	5'-TTAGGCAGGAACTGGAAGGG-3'
Ucp1	5'-TGTTGTCTTCAGGGCTGAGT-3'	5'-CTTCGGAAGTTGTCGGGTTC-3'
Cidea	5'-TGGTGGACACAGAGGAGTTC-3'	5'-TGCTTGCAGACTGGGACATA-3'
Zfp516	5'-CCCAGACTCTCTGAAAGCCA-3'	5'-GGAGAGCCTCCTTCACCTTT-3'
Elovl3	5'-CATCACCAGCCTGCAGATTC-3'	5'-AGTGTTCCGTTGTTGTGTGG-3'
Gapdh	5'-AATGTGTCCGTCGTGGATCT-3'	5'-AGACAACCTGGTCCTCAGTG-3'

Figure S1.

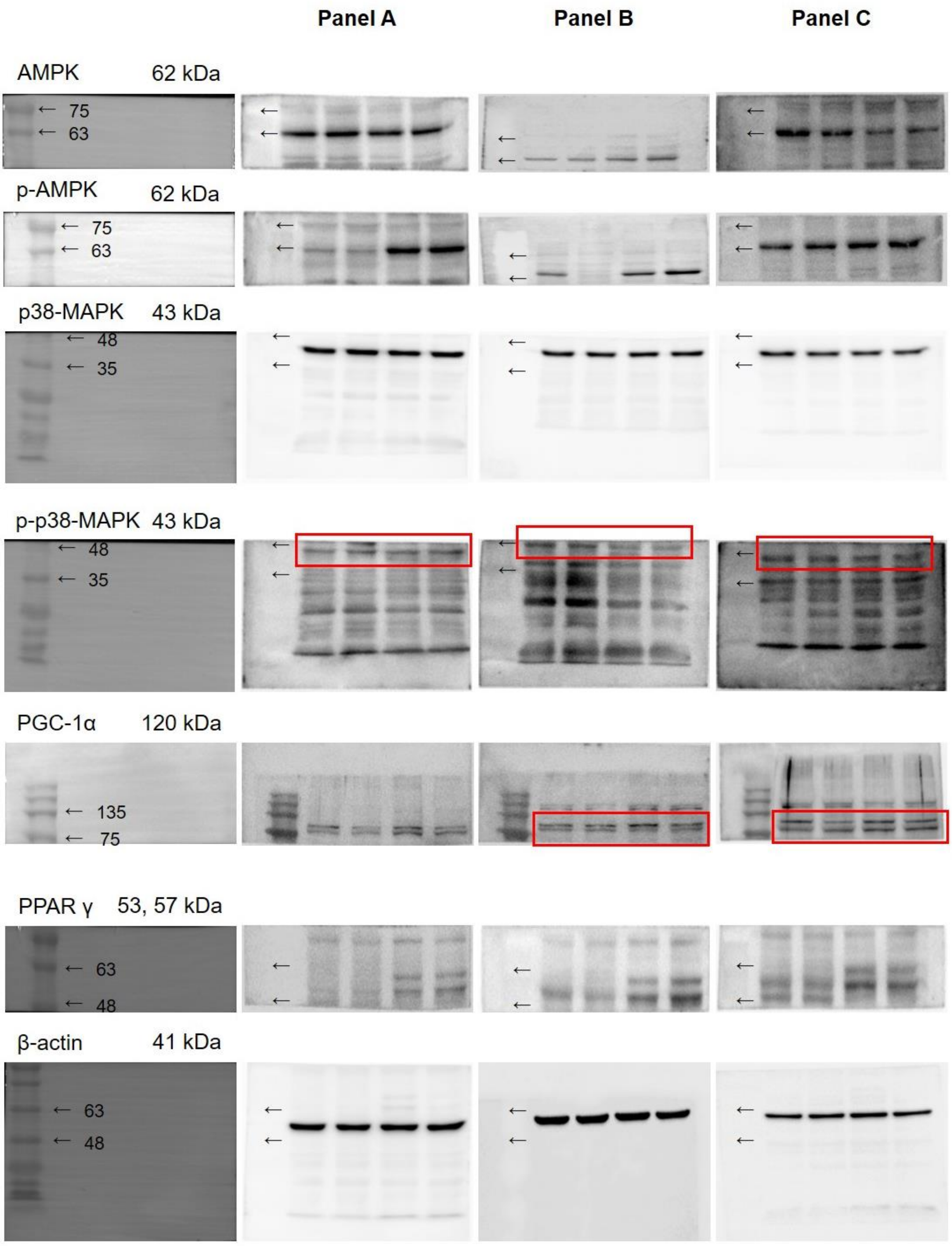

Figure S1. The original western blots for the effect of Acrp30 (adiponectin) treatment in undifferentiated and differentiated 3T3-L1 adipocyte cells. Each experiment was repeated three times.

Figure S2.

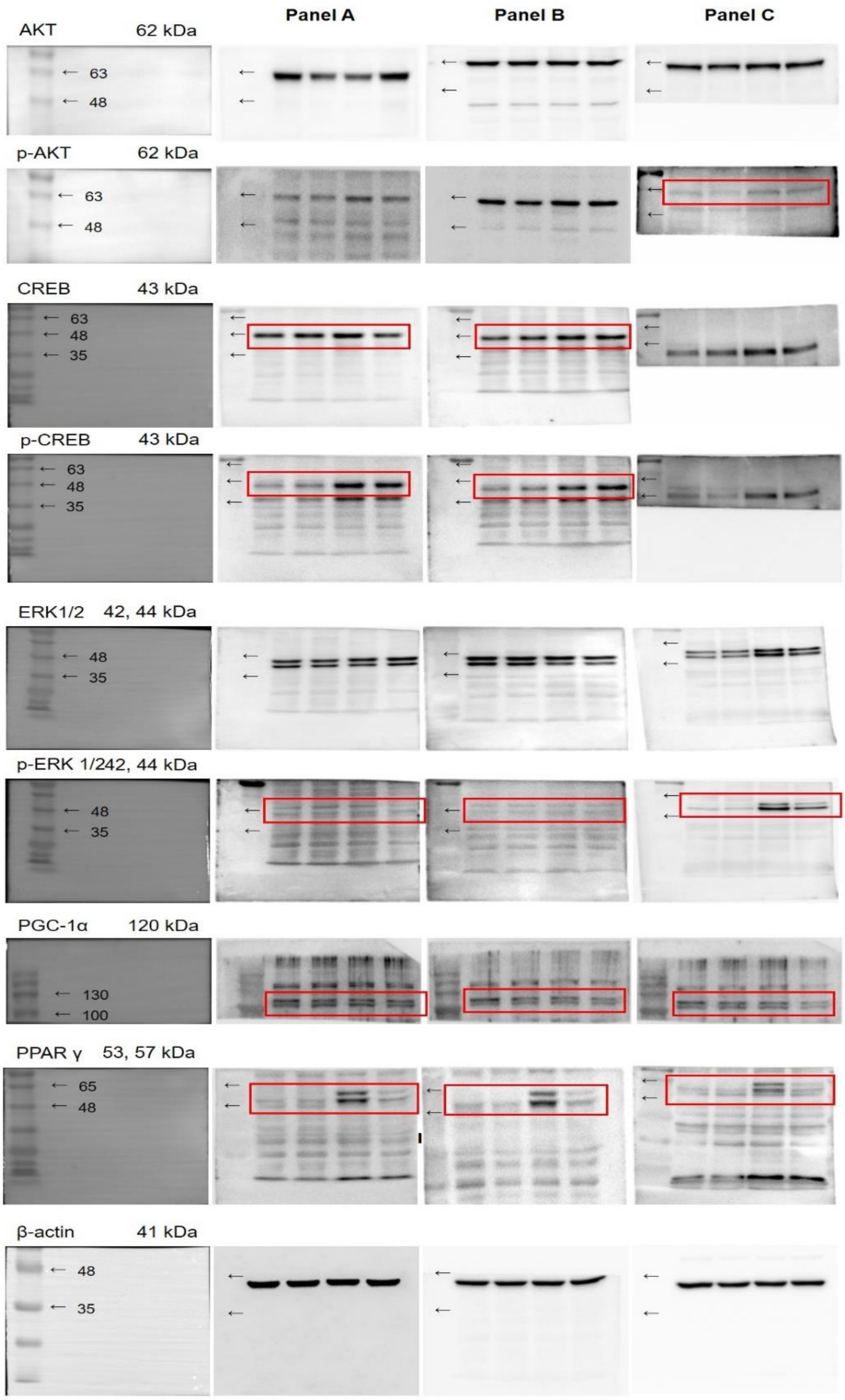

Figure S2. The original western blots for the effect of 7,8-DHF (BDNF mimetic) treatment in undifferentiated and differentiated 3T3-L1 adipocyte cells. Each experiment was repeated three times.
